# Chapter 16: Text Mining for Translational Bioinformatics

**DOI:** 10.1371/journal.pcbi.1003044

**Published:** 2013-04-25

**Authors:** K. Bretonnel Cohen, Lawrence E. Hunter

**Affiliations:** Computational Bioscience Program, University of Colorado School of Medicine, Aurora, Colorado, United States of America; Whitehead Institute, United States of America; University of Maryland, Baltimore County, United States of America

## Abstract

Text mining for translational bioinformatics is a new field with tremendous research potential. It is a subfield of biomedical natural language processing that concerns itself directly with the problem of relating basic biomedical research to clinical practice, and vice versa. Applications of text mining fall both into the category of T1 translational research—translating basic science results into new interventions—and T2 translational research, or translational research for public health. Potential use cases include better phenotyping of research subjects, and pharmacogenomic research. A variety of methods for evaluating text mining applications exist, including corpora, structured test suites, and post hoc judging. Two basic principles of linguistic structure are relevant for building text mining applications. One is that linguistic structure consists of multiple levels. The other is that every level of linguistic structure is characterized by ambiguity. There are two basic approaches to text mining: rule-based, also known as knowledge-based; and machine-learning-based, also known as statistical. Many systems are hybrids of the two approaches. Shared tasks have had a strong effect on the direction of the field. Like all translational bioinformatics software, text mining software for translational bioinformatics can be considered health-critical and should be subject to the strictest standards of quality assurance and software testing.

**What to Learn in This Chapter**

Text mining is an established field, but its application to translational bioinformatics is quite new and it presents myriad research opportunities. It is made difficult by the fact that natural (human) language, unlike computer language, is characterized at all levels by rampant ambiguity and variability. Important sub-tasks include gene name recognition, or finding mentions of gene names in text; gene normalization, or mapping mentions of genes in text to standard database identifiers; phenotype recognition, or finding mentions of phenotypes in text; and phenotype normalization, or mapping mentions of phenotypes to concepts in ontologies. Text mining for translational bioinformatics can necessitate dealing with two widely varying genres of text—published journal articles, and prose fields in electronic medical records. Research into the latter has been impeded for years by lack of public availability of data sets, but this has very recently changed and the field is poised for rapid advances. Like all translational bioinformatics software, text mining software for translational bioinformatics can be considered health-critical and should be subject to the strictest standards of quality assurance and software testing.

This article is part of the “Translational Bioinformatics” collection for *PLOS Computational Biology*.

## 1. Introduction

Text mining for translational bioinformatics is a new field with enormous research potential. It is a subfield of biomedical natural language processing (BioNLP) that concerns itself directly with the problem of relating basic biomedical research to clinical practice, and vice versa.

### 1.1 Use Cases

The foundational question in text mining for translational bioinformatics is what the use cases are. It is not immediately obvious how the questions that text mining for translational bioinformatics should try to answer are different from the questions that are approached in BioNLP in general. The answer lies at least in part in the nature of the specific kinds of information that text mining should try to gather, and in the uses to which that information is intended to be put. However, these probably only scratch the surface of the domain of text mining for translational bioinformatics, and the latter has yet to be clearly defined.

One step in the direction of a definition for use cases for text mining for translational bioinformatics is to determine classes of information found in clinical text that would be useful for basic biological scientists, and classes of information found in the basic science literature that would be of use to clinicians. This in itself would be a step away from the usual task definitions of BioNLP, which tend to focus either on finding biological information for biologists, or on finding clinical information for clinicians. However, it is likely that there is no single set of data that would fit the needs of biological scientists on the one hand or clinicians on the other, and that information needs will have to be defined on a bespoke basis for any given translational bioinformatics task.

One potential application is better phenotyping. Experimental experience indicates that strict phenotyping of patients improves the ability to find disease genes. When phenotyping is too broad, the genetic association may be obscured by variability in the patient population. An example of the advantage of strict phenotyping comes from the work of [1], [2]. They worked with patients with diagnoses of pulmonary fibrosis. However, having a diagnosis of pulmonary fibrosis in the medical record was not, in itself, a strict enough definition of the phenotype for their work [1]. They defined strict criteria for study inclusion and ensured that patients met the criteria through a number of methods, including manual review of the medical record. With their sharpened definition of the phenotype, they were able to identify 102 genes that were up-regulated and 89 genes that were down-regulated in the study group. This included Plunc (palate, lung and nasal epithelium associated), a gene not previously associated with pulmonary fibrosis. Automation of the step of manually reviewing medical records would potentially allow for the inclusion or exclusion of much larger populations of patients in similar studies.

Another use for text mining in translational bioinformatics is aiding in the preparation of Cochrane reviews and other meta-analyses of experimental studies. Again, text mining could be used to identify cohorts that should be included in the meta-analysis, as well as to determine P-values and other indicators of significance levels.

Most of the applications discussed here fall into the category of *T1 translational research*—translating basic science results into new interventions (http://grants.nih.gov/grants/guide/notice-files/NOT-AG-08-003.html). There are also applications in translational research for public health, also known as *T2 translational research* (op. cit.). This is true both in the case of mining information for public health experts and for the general public. For public health experts, there is a growing body of work on various factors affecting disease monitoring in electronic medical records, such as work by Chapman and colleagues on biosurveillance and disease and syndrome outbreak detection (e.g., [3], [4], among others). For the general public, simplifying technical texts can be helpful. [5] describes work in this area.

#### 1.1.1 The pharmacogenomics perspective

One area of research that has made some steps towards defining a use case for text mining is pharmacogenomics. An example of this is the PharmGKB database. Essential elements of their definition of pharmacogenomics text mining include finding relationships between genotypes, phenotypes, and drugs. As in the case of other applications that we will examine in this chapter, mining this information requires as a first step the ability to find mentions of the semantic types of interest when they are mentioned in text. These will be of increasing utility if they can be mapped to concepts in a controlled vocabulary. Each semantic type presents unique challenges. For example, finding information about genotypes requires finding mentions of genes (see Section 4.3 below), finding mentions of mutations and alleles, and mapping these to each other; finding mentions of drugs, which is more difficult than it is often assumed to be [6]; and finding mentions of phenotypes. The latter is especially difficult, since so many things can fit within the definition of “phenotype.” A phenotype is the entirety of observable characteristics of an organism [7]. The wide range and rapidly changing technologies for measuring observable features of patient phenotypes require the text mining user to be very specific about what observables they want to capture. For example, phenotypes can include any behavior, ranging from duration of mating dances in flies to alcohol-seeking in humans. They can also include any measurable physical characteristic, ranging from very “macro” characteristics such as hair color to very granular ones such as specific values for any of the myriad laboratory assays used in modern clinical medicine.

There is some evidence from the PharmGKB and the Comparative Toxicogenomics Database experiences that text mining can scale up processing in terms of the number of diseases studied and the number of gene-disease, drug-disease, and drug-gene associations discovered [8]. Furthermore, experiments with the PharmGKB database suggest that pharmacogenomics is currently more powerful than genomics for finding such associations and has reached the point of being ready for translation of research results to clinical practice [9].

#### 1.1.2 The i2b2 perspective

*Informatics for Integrating Biology and the Bedside* (i2b2) is a National Center for Biomedical Computing devoted to translational bioinformatics. It has included text mining within its scope of research areas. Towards this end, it has sponsored a number of shared tasks (see Section 5 below) on the subject of text mining. These give us some insight into i2b2's definition of use cases for text mining for translational bioinformatics. i2b2's focus has been on extracting information from free text in clinical records. Towards this end, i2b2 has sponsored shared tasks on deidentification of clinical documents, determining smoking status, detecting obesity and its comorbidities, medical problems, treatments, and tests. Note that there are no genomic components to this data.

### 1.2 Text Mining, Natural Language Processing, and Computational Linguistics

Text mining, natural language processing, and computational linguistics are often used more or less interchangeably, and indeed one can find papers on text mining and natural language processing at the annual meeting of the Association for Computational Linguistics, and papers from any of these categories at meetings for any of the other categories. However, technically speaking, some differences exist between them. Computational linguistics strictly defined deals with building computationally testable models of human linguistic behavior. Natural language processing has to do with building a wide range of applications that take natural language as their input. Text mining is more narrow than natural language processing, and deals with the construction of applications that provide a solution to a specific information need. For example, a syntactic analyzer would be an example of a natural language processing application; a text mining application might use that syntactic analyzer as part of the process for filling the very specific information need of finding information about protein-protein interactions. This chapter will include information about both natural language processing and text mining [10]–[12].

### 1.3 Evaluation Techniques and Evaluation Metrics in Text Mining

A variety of methods for evaluating text mining applications exist. They typically apply the same small family of metrics as figures of merit.

#### 1.3.1 Corpora

One paradigm of evaluation in text mining is based on the assumption that all evaluation should take place on naturally occurring texts. These texts are annotated with data or metadata about what constitutes the right answers for some task. For example, if the intended application to be tested is designed to locate mentions of gene names in free text, then the occurrence of every gene name in the text would be marked. The mark-up is known as *annotation*. (Note that this is a very different use of the word “annotation” from its use in the model organism database construction community.) The resulting set of annotated documents is known as a *corpus* (plural *corpora*). Given a corpus, an application is judged by its ability to replicate the set of annotations in the corpus. Some types of corpora are best built by linguists, e.g., those involving syntactic analysis, but there is abundant evidence that biomedical scientists can build good corpora if they follow best practices in corpus design (see e.g., [13]).

#### 1.3.2 Structured test suites

Structured test suites are built on the principles of software testing. They contain groups of inputs that are classified according to aspects of the input. For example, a test suite for applications that recognize gene names might contain sentences with gene names that end with numbers, that do not end with numbers, that consist of common English words, or that are identical to the names of diseases. Unlike a standard corpus, test suites may contain data that is manufactured for the purposes of the test suite. For example, a test suite for recognizing Gene Ontology terms [14] contains the term *cell migration*, but also the manufactured variant *migration of cells*. (Note that being manufactured does not imply being unrealistic.) Structured test suites have the major advantage of making it much more straightforward to evaluate both the strengths and the weaknesses of an application. For example, application of a structured test suite to an application for recognizing Gene Ontology terms made it clear that the application was incapable of recognizing terms that contain the word *in*. This was immediately obvious because the test suite contained sets of terms that contain function words, including a set of terms that all contain the word *in*. To duplicate this insight with a corpus would require assembling all errors, then hoping that the fact that no terms containing the word *in* were recognized jumped out at the analyst. In general, structured test suites should not be reflective of performance as measured by the standard metrics using a corpus, since the distribution of types of inputs in the test suite does not reflect the distribution of those types of inputs in naturally occurring data. However, it has been shown that structured test suites can be used to predict values of metrics for specific equivalence classes of data (inputs that should all be expected to test the same condition and produce the same result) [15]. We return to the use of test suites in Section 6.

#### 1.3.3 Post hoc judging

Sometimes preparation of corpora is impractical. For example, there may be too many inputs that need to be annotated. In these cases, post hoc judging is sometimes applied. That is, a program produces outputs, and then a human judges whether or not they are correct. This is especially commonly used when a large number of systems are being evaluated. In this case, the outputs of the systems can be pooled, and the most common outputs (i.e., the ones produced by the most systems) are selected for judging.

#### 1.3.4 Metrics

A small family of related metrics is usually used to evaluate text mining systems. *Accuracy*, or the number of correct answers divided by the total number of answers, is rarely used.

*Precision*. Precision is defined as the number of correct system outputs (“true positives,” or TP) divided by the total number of system outputs (the count of TP plus the “false positives” (FP) —erroneous system outputs). It is often compared loosely to specificity, but is actually more analogous to positive predictive value.
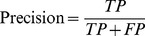



*Recall*. Recall is defined as the number of true positives divided by the total number of potential system outputs, i.e. true positives plus “false negatives” (FN) —things that should have been output by the system, but were not. This will differ from task type to task type. For example, in information retrieval (Section 4.1), it is the number of documents judged relevant divided by the total number of actual relevant documents. In named entity recognition of genes (Section 4.3), it is defined as the total number of correct gene names output by the system divided by the total number of gene names in the corpus.
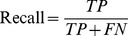



*Balanced F-measure*. The balanced F-measure attempts to reduce precision and recall to a single measure. It is calculated as the harmonic mean of precision and recall. It includes a parameter *β* that is usually set to one, giving precision and recall equal weight. Setting *β* greater than one weights precision more heavily. Setting *β* less than one weights recall more heavily.
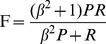



## 2. Linguistic Fundamentals

Building applications for text mining for translational bioinformatics is made easier by some understanding of the nature of linguistic structure. Two basic principles are relevant. One is that linguistic structure consists of multiple layers. The other is that every layer of linguistic structure is characterized by ambiguity.

All linguistic analyses in text mining are *descriptive* in nature. That is, they seek only to describe the nature of human linguistic productions, much as one might attempt to describe the multi-dimensional structure of a protein. Linguistic analyses are *not* prescriptive—that is, they do not attempt to prescribe or enforce standards for language use.

### 2.1 Layers of Linguistic Structure

The layers of linguistic structure vary somewhat between written and spoken language (although many are shared). We focus here on the layers that are relevant to written language, focusing particularly on scientific journal articles and on clinical documents.

#### 2.1.1 Document structure

The first layer of the structure of written documents that is relevant to text mining for translational bioinformatics is the structure of individual documents. In the case of journal articles, this consists first of all of the division of the document into discrete sections, typically in what is known as the IMRD model—an abstract, introduction, methods section, results section, discussion, and bibliography. Acknowledgments may be present, as well.

The ability to segment a document into these sections is important because different sections often require different processing techniques and because different sections should be focused on for different types of information. For example, methods sections are frequent sources of false positives for various semantic classes, which led researchers to ignore them in much early research. However, they are also fruitful sections for finding information about experimental methods, and as it has become clear that mining information about experimental methods is important to biologists [16], it has become clear that methods must be developed for dealing with methods sections. Abstracts have been shown to have different structural and content characteristics from article bodies [17]; most research to date has focused on abstracts, and it is clear that new approaches will be required to fully exploit the information in article bodies.

Segmenting and labeling document sections can be simple when documents are provided in XML and a DTD is available. However, this is often not the case; for instance, many documents are available for processing only in HTML format. In this situation, two topics exist: finding the boundaries of the sections, and labelling the sections. The latter is made more complicated by the fact that a surprising range of phrases are used to label the different sections of a scientific document. For example, the methods section may be called *Methods, Methods and Materials, Materials and Methods, Experimental Procedures, Patients and Methods, Study Design*, etc. Similar issues exist for structured abstracts; in the case of unstructured abstracts, it has been demonstrated that they can be segmented into sections using a generative technique [18].

Clinical documents present a far more complex set of challenges than even scientific journal articles. For one thing, there is a much wider range of clinical document types—admission notes, discharge summaries, radiology reports, pathology reports, office visit notes, etc. Hospitals frequently differ from each other in the types of documents that they use, as do individual physicians' practices. Furthermore, even within a given hospital, different physicians may structure the same document type differently. For example, just in the case of emergency room visit reports, one of the authors built a classification system that determined, for a given document, what specialty it would belong to (e.g., cardiology or pediatrics) if it had been generated by a specialist. He found that not only did each hospital require a different classification system, but different doctors within the same emergency room required different classifiers. [19] describes an iterative procedure for building a segmenter for a range of clinical document types.

Once the document has been segmented into sections, paragraphs must be identified. Here the segmentation task is typically easy, but ordering may present a problem. For example, it may not be clear where figure and table captions should be placed.

#### 2.1.2 Sentences

Once the document has been segmented into paragraphs, the paragraphs must be further segmented into sentences. Sentence segmentation is a surprisingly difficult task. Even for newswire text, it is difficult enough to constitute a substantial homework problem. For biomedical text, it is considerably more difficult. Two main difficulties arise. One is the fact that the function of periods is ambiguous—that is, a period may serve more than one function in a written text, such as marking the end of an abbreviation (*Dr.*), marking the individual letters of an abbreviation (*p.r.n.*), indicating the rational parts of real numbers (*3.14*), and so on. A period may even serve two functions, as for example when *etc.* is at the end of a sentence, in which case the period marks both the end of the abbreviation and the end of the sentence. Furthermore, some of the expected cues to sentence boundaries are absent in biomedical text. For example, in texts about molecular biology, it is possible for a sentence to begin with a lower-case letter when a mutant form of a gene is being mentioned. Various approaches have been taken to the sentence segmentation task. The KeX/PROPER system [20] uses a rule-based approach. The LingPipe system provides a popular machine-learning-based approach through its LingPipe API. Its model is built on PubMed/MEDLINE documents and works well for journal articles, but it is not likely to work well for clinical text (although this has not been evaluated). In clinical documents, it is often difficult to define any notion of “sentence” at all.

#### 2.1.3 Tokens

Written sentences are built up of tokens. Tokens include words, but also punctuation marks, in cases where those punctuation marks should be separated from words that they are attached to. The process of segmenting a sentence into tokens is known as *tokenization*. For example, consider the simple case of periods. When a period marks the end of a sentence, it should be separated from the word that it is attached to. *regulation*. will not be found in any biomedical dictionary, but *regulation* will. However, in many other instances, such as when it is part of an abbreviation or a number, it should not be separated. The case of hyphens is even more difficult. Hyphens may have several functions in biomedical text. If they indicate the absence of a symptom (e.g., *-fever*), they should probably be separated, since they have their own meaning, indicating the absence of the symptom. On the other hand, they should remain in place when separating parts of a word, such as *up-regulate*.

The status of tokenization in building pipelines of text mining applications is complicated. It may be the case that a component early in the pipeline requires tokenized text, while a component later in the pipeline requires untokenized text. Also, many applications have a built-in tokenizer, and conflicts between different tokenization strategies may cause conflicts in later analytical strategies.

#### 2.1.4 Stems and lemmata

For some applications, it is advantageous to reduce words to stems or lemmata. Stems are normalized forms of words that reduce all inflected forms to the same string. They are not necessarily actual words themselves—for example, the stem of *city* and *cities* is *citi*, which is not a word in the English language. Their utility comes in applications that benefit from this kind of normalization without needing to know exactly which words are the roots—primarily machine-learning-based applications.

The term *lemma* (plural *lemmata*) is overloaded. It can mean the root word that represents a set of related words. For example, the lemma of the set {*phosophorylate, phosphorylates, phosphorylated, phosphorylating*} is *phosphorylate*. Note that in this case, we have an actual word. *Lemma* can also mean the set of words that can instantiate a particular root word form; on this meaning, the lemma of *phosphorylate* is {*phosphorylate, phosphorylates, phosphorylated, phosphorylating*}. Lemmas have a clear advantage of stems for some applications. However, while it is always possible to determine the stem of a word (typically using a rule-based approach, such as the Porter stemmer [21], it is not always possible to determine the lemma of a word automatically. The BioLemmatizer [22] is a recently released tool that shows high performance on the lemmatization task.

#### 2.1.5 Part of speech

It is often useful to know the part of speech, technically known as *lexical category*, of the tokens in a sentence. However, the notion of part of speech is very different in linguistic analysis than in the elementary school conception, and text mining systems typically make use of about eighty parts of speech, rather than the eight or so that are taught in school. We go from eight to eighty primarily by subdividing parts of speech further than the traditional categories, but also by adding new ones, such as parts of speech of sentence-medial and sentence-final punctuation. Parts of speech are typically assigned to tokens by applications called *part of speech taggers*. Part of speech tagging is made difficult by the fact that many words are ambiguous as to their part of speech. For example, in medical text, the word *cold* can be an adjective or it can be a reference to a medical condition. A word can have several parts of speech, e.g., *still*. A variety of part of speech taggers that are specialized for biomedical text exist, including MedPOST [23], LingPipe, and the GENIA tagger [24].

#### 2.1.6 Syntactic structure

The *syntactic structure* of a sentence is the way in which the phrases of the sentence relate to each other. For example, in the article title *Visualization of bacterial glycocalyx with a scanning electron microscope* (PMID 9520897), the phrase *with a scanning electron microscope* is associated with *visualization*, not with *bacterial glycocalyx*. Automatic syntactic analysis is made difficult by the existence of massive ambiguity. For example, while one possible interpretation of that title is that the visualization is done with a scanning electron microscope, another possible interpretation is that the bacterial glycocalyx has a scanning electron microscope. (Consider the analogous famous example *I saw the man with the binoculars*, where one possible interpretation is that I used the bionoculars to visualize the man, whereas another possible interpretation is that I saw a man and that man had some binoculars.) It is very easy for humans to determine which interpretation of the article title is correct. However, it is very difficult for computers to make this determination. There are *many* varieties of syntactic ambiguity, and it is likely that any nontrivial sentence contains at least one.

Syntactic analysis is known as *parsing*. The traditional approach to automated syntactic analysis attempts to discover the phrasal structure of a sentence, as described above. A new approach called *dependency parsing* focuses instead on relationships between individual words. It is thought to better reflect the semantics of a sentence, and is currently popular in BioNLP.

Along with determining the phrasal or dependency structure of a sentence, some parsers also make limited attempts to label the syntactic functions, such as *subject* and *object*, of parts of a sentence.

### 2.2 The Nature of Linguistic Rules

When we think of linguistic rules, we are most likely to think of the rules that we learn in school that impose arbitrary norms on language usage, such as *Say “you and I”, not “you and me”*, or *a preposition is a bad thing with which to end a sentence*. These are known as *prescriptive rules*. Text mining never deals with prescriptive rules. Rather, it always deals with *descriptive rules*. Descriptive rules *describe* the parts of the language and the ways in which they can combine, without any implied judgement as to whether they are “good” or “bad.” For example, a linguistic rule might specify that certain classes of verbs can be converted to nouns by adding *-tion* to their end, or that when a passive form of a verb is used, the subject can be omitted.

## 3. The Two Families of Approaches: Rule-Based and Learning-Based

There are two basic approaches to text mining: rule-based, also known as knowledge-based, and machine-learning-based, also known as statistical.

*Rule-based approaches to text mining* are based on the application of rules, typically manually constructed, to linguistic inputs. For example, a rule-based approach to syntactic analysis might postulate that given a string like *phosphorylation of MAPK by MAPKK*, the phrase that follows the word *by* is the doer of the phosphorylation, and the phrase that follows the word *of* is the undergoer of the phosphorylation. Or, a rule-based approach might specify that in the pattern *A X noun* the *X* is an adjective, while in the pattern *The adjective X verb* the X is a noun, allowing us to differentiate between the word *cold* as an adjective in the former case and as a medical condition in the latter case. Rule-based solutions can be constructed for all levels of linguistic analysis.

*Machine-learning-based approaches to text mining* are based on an initial step of feeding the system a set of data that is labelled with the correct answers, be they parts of speech for tokens or the locations of gene names in text. The job of the system is then to figure out cues that indicate which of the ambiguous analyses should be applied. For instance, a system for document classification may learn that if a document contains the word *murine*, then it is likely to be of interest to researchers who are interested in mice. Many different algorithms for machine learning exist, but the key to a successful system is the set of features that are used to perform the classification. For example, a part of speech tagger may use the apparent parts of speech of the two preceding words as a feature for deciding the part of speech of a third word.

It is often claimed that machine learning systems can be built more quickly than rule-based systems due to the time that it takes to build rules manually. However, building feature extractors is time-consuming, and building the labelled “training” data with the right answers is much more so. There is no empirical support for the claim that learning-based systems can be built more quickly than rule-based systems. Furthermore, it is frequently the case that putative learning-based systems actually apply rules in pre- or post-processing steps, making them hybrid systems.

## 4. Text Mining Tasks

In Section 2.1, we discussed elements of linguistic analysis. These analytical tasks are carried out in support of some higher-level text mining tasks. Many types of text mining tasks exist. We will discuss only the most common ones here, but a partial list includes:

- Information retrieval
- Document classification
- Named entity recognition
- Named entity normalization
- Relation or information extraction
- Question-answering
- Summarization

### 4.1 Information Retrieval

*Information retrieval* is the task of, given an information need and a set of documents, finding the documents that are relevant to filling that information need. PubMed/MEDLINE is an example of a biomedical information retrieval system for scientific journal articles; Google is an information retrieval system for web pages. Early information retrieval assumed that all documents were classified with some code and typically required the assistance of a librarian to determine the appropriate code of interest. *Keyword*-based retrieval, in which the user enters a set of words that a relevant text would be expected to contain and the content of the texts in the set of documents are searched for those words, was a revolution made possible by the introduction of computers and electronic forms of documents in the hospital or research environment. The naive approach to keyword-based retrieval simply checks for the presence or absence of the words in the query, known as boolean search. Modern approaches use relatively simple mathematical techniques to determine (a) the relative importance of words in the query in deciding whether or not a document is relevant—the assumption here is that not all words are equally important—and (b) how well a given word reflects the actual relevance of a given document to the query. For example, we can determine, given a count of how often the words *hypoperfusion* and *kidney* occur in the set of documents as a whole, that if we are looking for documents about kidney hypoperfusion, we should give more weight to the rarer of the two words; given a count of how often the words *kidney* and *hypoperfusion* occur in two documents, we can determine which of the two documents is most relevant to the query.

### 4.2 Document Classification

*Document classification* is the task of classifying a document as a member of one or more categories. In a typical document classification workflow, one is supplied with a stream of documents, and each one requires classification. This differs from the information retrieval situation, in which information needs are typically ad hoc. For example, curators of a model organism database may require journal articles to be classified as to whether or not they are relevant for further examination. Other classification tasks motivated by curation have been classifying journal articles as to whether or not they are about embryogenesis. Document classification typically uses very simple feature sets, such as the presence or absence of the words from the training data. When this is the only feature, it is known as a “bag of words” representation. However, it has also been found useful to use more abstract, conceptual features. For example, [25] found the presence or absence of mentions of mouse strains to be a useful feature, regardless of the identity of the particular strain.

### 4.3 Named Entity Recognition

*Named entity recognition* is the task of finding mentions of specific semantic classes in a text. In general language processing, the most heavily studied semantic classes have been persons, places, and organizations—thus, the term “named entity.” In genomic BioNLP, the most heavily studied semantic class has been gene and protein names. However, other semantic classes have been studied as well, including cell lines and cell types. In clinical NLP, the range of semantic classes is wider, encompassing a large number of types included in the Unified Medical Language System [26]. The UMLS includes a “Metathesaurus” which combines a large number of clinically and biologically relevant controlled vocabularies, comprising many semantic classes. In the clinical domain, there is an “industry standard” tool for named entity recognition, called MetaMap [27], [28]. Biological named entity recognition remains a subject of current research. Machine learning methods predominate. Feature sets generally include typographical features of a token—e.g., having mixed-case letters or not, containing a hyphen or not, ending with a numeral or not, etc. —as well as features of the surrounding tokens.

Early results in named entity recognition were consistent with the hypothesis that this task could not be achieved by simply starting with a “dictionary” of gene names and looking for those gene names in text. At least three problems were immediately evident with this approach—the fact that new gene names are coined constantly, the fact that a number of gene names are homographs of common English words, and the fact that many genes have names or synonyms that are unhelpful, such as *putative oxidoreductase* (Entrez Gene ID 6393330). However, recent evidence has suggested that dictionary-based approaches can achieve moderate success if the dictionary and the data to be processed are subjected to extensive preprocessing [29] or post-hoc filtering, e.g., by the success or failure of a subsequent gene normalization step (see Section 4.4 [30]).

### 4.4 Named Entity Normalization

*Named entity normalization* is the process of taking a mention of a named entity in free text and returning a specific database identifier that it refers to. In the biological domain, this has been studied most extensively in the case of genes and proteins, and the corresponding task is known as *gene normalization*. In the clinical domain, it has been approached simultaneously with named entity recognition, again using the MetaMap application (see Section 4.3). There are two major problems in gene normalization. The first is that many species have genes with the same name. For example, the BRCA1 gene is found in an enormous number of animals. Thus, finding the appropriate gene identifier requires knowing the species under discussion, which is a research problem in itself. The other problem is that a single species may have multiple genes with the same name. For example, humans have five genes named *TRP-1*. Gene normalization is often approached as a problem in *word sense disambiguation*, the task of deciding which dictionary entry a given text string refers to (e.g., the *cold* example referred to above). A popular approach to this utilizes knowledge about the gene and the context in which the gene is mentioned. For example, the SUMMARY fields of the candidate genes might be used as a source of words that indicate what we know about the gene. Then, if we see the words *cation* and *channel* in the text surrounding the gene name, we should expect that we have an instance of the *TRP1* with Entrez Gene ID 7220, while if we see the word *proline*, we should suspect that we have an instance of the *TRP1* with Entrez Gene ID 189930. Approaches might vary with respect to what they use as the knowledge source (e.g., Entrez Gene SUMMARY fields, Entrez Gene PRODUCT fields, the contents of publications linked to the Entrez Gene entry), and what they consider the context of the gene mention, e.g., the sentence, the surrounding sentences, the entire abstract, etc.

### 4.5 Relation or Information Extraction

*Information extraction*, or more recently *relation extraction*, is the process of mining very specific types of facts from text. Information extraction systems are by definition restricted to a very specific type of information. For example, a typical genomic information extraction system might extract assertions about protein-protein interactions, or a clinical information extraction system might mine assertions about relationships between diseases and their treatments. Most systems target binary relations, such as the ones just described. However, more ambitious systems have extracted relationships with as many as four participants. One system [31] targeted protein transport relations, with a four-way relationship that included the transporting protein, the transported protein, the beginning location of the transported protein, and the destination.

Rule-based approaches use typical sentence patterns. These may consist of text literals or may involve syntactic analyses [32]. Learning-based approaches have classically used bag-of-words representations (see Section 4.2), but more recent approaches have had success using features taken from syntactic analysis, particularly dependency parsing [33].

### 4.6 Question-Answering

*Question-answering* is the task of taking a question and a source of information as input and returning an answer. Early approaches to question-answering assumed that the source of information was a database, but modern approaches assume that the answer exists in some PubMed/MEDLINE document or (for non-biomedical applications) in some web page. Question-answering differs from information retrieval in that the goal is to return a specific answer, not a document containing the answer. It differs from information extraction in that it is meant to allow for ad hoc queries, while information extraction focuses on very specific information needs. Question-answering typically involves determining the type of answer that is expected (a time? a location? a person?), formulating a query that will return documents containing the answer, and then finding the answer within the documents that are returned.

Various types of questions have varying degrees of difficulty. The best results are achieved for so-called “factoid” questions, such as *where are lipid rafts located?*, while “why” questions are very difficult. In the biomedical domain, definition questions have been extensively studied [34]–[36]. The medical domain presents some unique challenges. For example, questions beginning with *when* might require times as their answer (e.g., *when does blastocyst formation occur in humans?*, but also may require very different sorts of answers, e.g.,*when should antibiotics be given for a sore throat?* [37]. A shared task in 2005 involved a variety of types of genomic questions adhering to specific templates (and thus overlapping with information extraction), such as *what is the biological impact of a mutation in the gene X?*.

### 4.7 Summarization

*Summarization* is the task of taking a document or set of documents as input and returning a shorter text that conveys the information in the longer text(s). There is a great need for this capability in the biomedical domain—a search in PubMed/MEDLINE for the gene p53 returns 56,464 publications as of the date of writing.

In the medical domain, summarization has been applied to clinical notes, journal articles, and a variety of other input types. For example, one system, MITRE'S MiTAP, does multi-document summarization of epidemiological reports, newswire feeds, email, online news, television news, and radio news to detect disease outbreaks.

In the genomics domain, there have been three major areas of summarization research. One has been the automatic generation of GeneRIFs. GeneRIFs are short text snippets, less than 255 characters in length, associated with specific Entrez Gene entries. Typically they are manually cut-and-pasted from article abstracts. Lu et al. developed a method for finding them automatically using a variant of the Edmundsonian paradigm, a classic approach to single-document summarization [38], [39]. In the Edmundsonian paradigm, sentences in a document are given points according to a relatively simple set of features, including position in the document, presence of “cue words” (words that indicate that a document is a good summary sentence), and absence of “stigma words” (words that indicate that a sentence is not likely to be a good summary sentence).

Another summarization problem is finding the best sentence for asserting a protein-protein interaction. This task was made popular by the BioCreative shared task. The idea is to boil down a set of articles to the single sentence that best gives evidence that the interaction occurs. Again, simple features work well, such as looking for references to figures or tables [40].

Finally, a small body of work on the generation of SUMMARY fields has been seen. More sophisticated measures have been applied here, such as the PageRank algorithm [41].

## 5. Shared Tasks

The natural language processing community has a long history of evaluating applications through the shared task paradigm. Similar to CASP, a *shared task* involves agreeing on a task definition, a data set, and a scoring mechanism. In biomedical text mining, shared tasks have had a strong effect on the direction of the field. There have been both clinically oriented and genomically oriented shared tasks.

In the clinical domain, the 2007 NLP Challenge [42] involved assigning ICD9-CM codes to radiology reports of chest x-rays and renal procedures. Also in the clinical domain, i2b2 has sponsored a number of shared tasks, described in Section 1.1.2. (At the time of writing, the National Institute of Standards and Technology is preparing a shared task involving electronic medical records under the aegis of the annual Text Retrieval Conference. The task definition is not yet defined.)

In the genomics domain, the predominant shared tasks have been the BioCreative shared tasks and a five-year series of tasks in a special genomics track of the Text Retrieval Conference [43]. Some of the tasks were directly relevant to translational bioinformatics. The tasks varied from year to year and included information retrieval (Section 4.1), production of GeneRIFs (Section 4.7), document classification (Section 4.2), and question-answering (Section 4.6). A topic that was frequently investigated by participants was the contribution of controlled vocabularies to performance on text mining tasks. Results were equivocal; it was found that they could occasionally increase performance, but only when used intelligently, e.g., with appropriate preprocessing or filtering of items in the terminologies—blind use of vocabulary resources does not improve performance.

The BioCreative series of shared tasks has been oriented more towards model organism database curation than towards translational bioinformatics, but some of the subtasks that were involved are of utility in translational bioinformatics. BioCreative tasks have included gene name recognition in text (Section 4.3), mining information about relationships between genes and their functions (Section 4.5), mining information about protein-protein interactions (Section 4.5), information retrieval (Section 4.1), and relating mentions of genes in text to database entries in Entrez Gene and SWISSPROT (Section 4.4).

## 6. Software Engineering for Text Mining

Like all translational bioinformatics software, text mining software for translational bioinformatics can be considered health-critical and should be subject to the strictest standards of quality assurance and software testing. General software testing is covered in such standard books as [44]. The special requirements of software testing for natural language processing applications are not covered in the standard books on software testing, but a small but growing body of literature discusses the special issues that arise here. There are two basic paradigms for evaluating text mining applications. The standard paradigm involves running large corpora through the application and determining the F-measure achieved. However, this approach is not satisfactory for quality assurance and software testing. It is good for achieving overall estimates of performance, but does a poor job of indicating what the application is good at and what it is bad at. For this task, structured test suites and application of the general principles of software testing are much more appropriate. Structured test suites are discussed in Section 1.3.2. It is helpful to consult with a descriptive linguist when designing test suites for assessing an application's ability to handle linguistic phenomena. [15] and [14] describe basic principles for constructing test suites for linguistic phenomena by applying the techniques of software testing and of descriptive linguistics. The former includes a methodology for the automatic generation of test suites of arbitrary size and complexity. [45] presents a quantitative examination of the effectiveness of corpora versus structured test suites for software testing, and demonstrates that structured test suites achieve better code coverage (percentage of code that is executed during the test phase—bugs cannot be discovered in code that is not executed) than corpora, and also offer a significant advantage in terms of time and efficiency. They found that a structured test suite that achieved higher code coverage than a 3.9 million word corpus could be run in about 11 seconds, while it took about four and a half hours to process the corpus. [46] discusses the application of the software engineering concept of the “fault model,” informed by insights from linguistics, to discovering a serious error in their ontology linking tool.

User interface assessment requires special techniques not found in other areas of software testing for natural language processing. User interface testing has been most heavily studied in the case of literature search interfaces. Here the work of [47], [48] is most useful, and can serve as a tutorial on interface evaluation.

## 7. Exercises

1. Obtain a copy of a patient record collection from the i2b2 National Center for Biomedical Computing (see e.g., [49]). Download the MetaMap application or API and run it over a set of ten discharge summaries. Use Google to find the current links for the i2b2 data sets and for downloading MetaMap. Note that using the MetaMap application will require writing code to extract results from the MetaMap output file, while using the API will require writing your own application. Which outputs might you consider to identify phenotypes that could be relevant for your research interests?
2. Obtain a collection of 1,000 PubMed abstracts by querying with the terms *gene* and *mutation* and downloading the 1,000 most recent. Run the EMU mutation extractor (http://bioinf.umbc.edu/EMU/ftp) or a similar tool on them. What genotypes can you identify in the output?
3. A researcher has a collection of 10,000 documents. She wants to retrieve all documents relevant to pulmonary hypertension. The collection contains 250 documents that are relevant to pulmonary hypertension. An information retrieval program written by a colleague returns 100 documents. 80 of these are actually relevant to pulmonary hypertension. What is the precision, recall, and F-measure for this system?
4. Explain the difference between *descriptive linguistic rules* and *prescriptive linguistic rules*. Be sure to say which type text mining is concerned with.

Answers to the Exercises can be found in Text S1.

**Table 1:** Some knowledge sources for biomedical natural language processing.

Informatics for Integrating Biology and the Bedside (i2b2 - https://www.i2b2.org/)	National Center for Biomedical Computing with focus on translational research that facilitates and proves data sets for clinical natural language processing research
Gene Ontology (https://www.geneontology.org)	Controlled vocabulary with relationships including partonymy and inheritance, designed for describing gene functions, broadly construed
Entrez Gene (https://www.ncbi.nlm.nih.gov/gene)	Source for gene names, symbols, and synonyms; also the source for GeneRIFs and SUMMARY fields
PubMed/MEDLINE (https://www.ncbi.nlm.nih.gov/pubmed)	The National Library of Medicine's database of abstracts of biomedical publications (MEDLINE) and search interface for accessing them (PubMed)
Unified Medical Language System (https://www.nlm.nih.gov/research/umls/)	Large lexical and conceptual resource, including the UMLS Metathesaurus, which aggregates a large number of biomedical and some genomic vocabularies
SWISSPROT (https://www.uniprot.org/)	Database of information about proteins with literature references, useful as a gold standard
PharmGKB (https://www.pharmgkb.org/)	Database of relationships between a number of clinical, genomic, and other entities with literature references, useful as a gold standard
Comparative Toxicogenomics Database (https://ctdbase.org/)	Database of relationships between genes, diseases, and chemicals, with literature references, useful as a gold standard

Various terminological resources, data sources, and gold-standard databases for biomedical natural language processing.

**Further Reading**

[50] is a book-length treatment of biomedical natural language processing, oriented towards readers with some background in NLP. [51] takes the perspective of model organism database curation as the primary motivating task for text mining. It includes a review of the ten most important papers and resources for BioNLP. [52] is an excellent introduction to text mining in general. [10] is the standard textbook on natural language processing.

There are a number of seminal papers on biomedical text mining besides those already cited in the text. These include [20], [53]–[57].

Table 1 lists a variety of terminological resources, data sources, and gold-standard databases for biomedical natural language processing.

## Supporting Information

Text S1 Answers to Exercises.
(DOCX)

## References

[1] Steele MP, Speer MC, Loyd JE, Brown KK, Herron A, et al (2005) Clinical and pathologic features of familial interstitial pneumonia. Am J Respir Crit Care Med 172: 1146–1152. 16109978 10.1164/rccm.200408-1104OC PMC2718398

[2] Boon K, Bailey N, Yang J, Steel M, Groshong S, et al (2009) Molecular phenotypes distinguish patients with relatively stable from progressive idiopathic pulmonary fibrosis (ipf). PLoS ONE 4: e5134 doi:10.1371/journal.pone.0005134. 19347046 10.1371/journal.pone.0005134 PMC2661376

[3] Chapman W, Dowling J, Wagner M (2004) Fever detection from free-text clinical records for biosurveillance. J Biomed Inform 37: 120–127. 15120658 10.1016/j.jbi.2004.03.002 PMC7128853

[4] Chapman W, Dowling J (2007) Can chief complaints detect febrile syndromic patients? Journal of Advances in Disease Surveillance 3.

[5] Elhadad N (2006) User-sensitive text summarization: application to the medical domain [Ph.D. thesis]. New York: Columbia University.

[6] Uzuner O, Solti I, Cadag E (2010) Extracting medication information from clinical text. J Am Med Inform Assoc 17: 514–518. 20819854 10.1136/jamia.2010.003947 PMC2995677

[7] Hunter LE (2009) The processes of life: an introduction to molecular biology. Cambridge (MA): MIT Press.

[8] Wiegers TC, Davis AP, Cohen KB, Hirschman L, Mattingly CJ (2009) Text mining and manual curation of chemical-gene-disease networks for the Comparative Toxicogenomics Database (CTD). BMC Bioinformatics 10: 326. 19814812 10.1186/1471-2105-10-326 PMC2768719

[9] Altman RB (2011) Pharmacogenomics: “noninferiority” is sufficient for initial implementation. Clin Pharmacol Ther 89: 348–350. 21326263 10.1038/clpt.2010.310

[10] Jurafsky D, Martin JH (2008) Speech and language processing: an introduction to natural language processing, computational linguistics, and speech recognition. Pearson Prentice Hall

[11] Manning C, Schuetze H (1999) Foundations of statistical natural language processing. Cambridge (MA): MIT Press.

[12] Jackson P, Moulinier I (2002) Natural language processing for online applications: text retrieval, extraction, and categorization. 2nd edition. John Benjamins Publishing Company

[13] Cohen KB, Fox L, Ogren PV, Hunter L (2005) Empirical data on corpus design and usage in biomedical natural language processing. In: AMIA 2005 symposium proceedings. pp. 156–160. PMC1560643 16779021

[14] Cohen KB, Roeder C, Jr WAB, Hunter L, Verspoor K (2010) Test suite design for biomedical ontology concept recognition systems. In: Proceedings of the Language Resources and Evaluation Conference.

[15] Cohen KB, Tanabe L, Kinoshita S, Hunter L (2004) A resource for constructing customized test suites for molecular biology entity identification systems. In: HLT-NAACL 2004 Workshop: BioLINK 2004, Linking Biological Literature, Ontologies and Databases. Association for Computational Linguistics, pp. 1–8.

[16] Krallinger M, Morgan A, Smith L, Leitner F, Tanabe L, et al (2008) The BioCreative II – critical assessment for information extraction in biology challenge. Genome Biol 9.

[17] Cohen KB, Johnson HL, Verspoor K, Roeder C, Hunter LE (2010) The structural and content aspects of abstracts versus bodies of full text journal articles are different. BMC Bioinformatics 11: 492. 20920264 10.1186/1471-2105-11-492 PMC3098079

[18] Lin J, Karakos D, Demner-Fushman D, Khudanpur S (2006) Generative content models for struc-tural analysis of medical abstracts. In: Proceedings of the HLT-NAACL BioNLP Workshop on Linking Natural Language and Biology. New York, New York: Association for Computational Linguistics. pp. 65–72.

[19] Demner-Fushman D, Abhyankar S, Jimeno-Yepes A, Loane R, Rance B, et al.. (2011) A knowledge-based approach to medical records retrieval. In: Proceedings of TREC 2011.

[20] Fukuda K, Tamura A, Tsunoda T, Takagi T (1998) Toward information extraction: identifying protein names from biological papers. In: Pac Symp Biocomput. pp. 707–718. 9697224

[21] Porter MF (1980) An algorithm for suffix stripping. Program 14: 130–137.

[22] Liu H, Christiansen T, Baumgartner WA Jr, Verspoor K (2012) BioLemmatizer: a lemmatization tool for morphological processing of biomedical text. J Biomed Semantics 3: 3. 22464129 10.1186/2041-1480-3-3 PMC3359276

[23] Smith L, Rindesch T, Wilbur WJ (2004) Medpost: A part-of-speech tagger for biomedical text. Bioinformatics 20: 2320–2321. 15073016 10.1093/bioinformatics/bth227

[24] Tsuruoka Y, Tateishi Y, Kim JD, Ohta T, Mcnaught J, et al.. (2005) Developing a robust part-of-speech tagger for biomedical text. In: Proceedings of the 10th Panhellenic Conference on Informatics. pp. 382–392.

[25] Caporaso JG, Baumgartner WA Jr, Cohen KB, Johnson HL, Paquette J, et al.. (2005) Concept recognition and the TREC Genomics tasks. In: The Fourteenth Text REtrieval Conference (TREC 2005) Proceedings.

[26] Lindberg D, Humphreys B, Mccray A (1993) The Unified Medical Language System. Methods Inf Med 32: 281–291. 8412823 10.1055/s-0038-1634945 PMC6693515

[27] Aronson A (2001) Effective mapping of biomedical text to the UMLS Metathesaurus: The MetaMap program. In: Proc AMIA 2001. pp. 17–21. PMC2243666 11825149

[28] Aronson AR, Lang FM (2010) An overview of MetaMap: historical perspective and recent advances. J Am Med Inform Assoc 17: 229–236. 20442139 10.1136/jamia.2009.002733 PMC2995713

[29] Hanisch D, Fundel K, Mevissen HT, Zimmer R, Fluck J (2005) ProMiner: rule-based protein and gene entity recognition. BMC Bioinformatics 6 Suppl 1: S14. 10.1186/1471-2105-6-S1-S14 PMC1869006 15960826

[30] Verspoor K, Roeder C, Johnson HL, Cohen KB, Baumgartner WA Jr, et al (2010) Exploring species-based strategies for gene normalization. IEEE/ACM Trans Comput Biol Bioinform 7: 462–471. 20671318 10.1109/TCBB.2010.48 PMC2929766

[31] Hunter L, Lu Z, Firby J, Baumgartner WA Jr, Johnson HL, et al (2008) OpenDMAP: An open-source, ontology-driven concept analysis engine, with applications to capturing knowledge regarding pro-tein transport, protein interactions and cell-specific gene expression. BMC Bioinformatics 9: 78. 18237434 10.1186/1471-2105-9-78 PMC2275248

[32] Kilicoglu H, Bergler S (2009) Syntactic dependency based heuristics for biological event extraction. In: Proceedings of the BioNLP 2009 Workshop Companion Volume for Shared Task. Boulder, Colorado: Association for Computational Linguistics. pp. 119–127.

[33] Kim JD, Ohta T, Pyysalo S, Kano Y, Tsujii J (2009) Overview of BioNLP'09 shared task on event extraction. In: BioNLP 2009 Companion Volume: Shared Task on Entity Extraction. pp. 1–9.

[34] Lin J, Demner-Fushman D (2005) Automatically evaluating answers to definition questions. In: Proceedings of the 2005 Human Language Technology Conference and Conference on Empirical Methods in Natural Language Processing (HLT/EMNLP 2005). pp. 931–938.

[35] Yu H, Wei Y (2006) The semantics of a definiendum constrains both the lexical semantics and the lexicosyntactic patterns in the definiens. In: HTL-NAACL BioNLP Workshop: Linking Natural Language Processing and Biology: Towards Deeper Biological Literature Analysis. ACL, pp. 1–8.

[36] Yu H, Lee M, Kaufman D, Ely J, Osheroff J, et al (2007) Development, implementation, and a cognitive evaluation of a definitional question answering system for physicians. J Biomed Inform 40: 236–251. 17462961 10.1016/j.jbi.2007.03.002

[37] Zweigenbaum P (2003) Question answering in biomedicine. In: Proceedings of the workshop on natural language processing for question answering. pp. 1–4.

[38] Lu Z, Cohen BK, Hunter L (2006) Finding GeneRIFs via Gene Ontology annotations. In: PSB 2006. pp. 52–63. PMC2652876 17094227

[39] Lu Z, Cohen KB, Hunter L (2007) GeneRIF quality assurance as summary revision. In: Pacific Symposium on Biocomputing. 10.1142/9789812772435_0026 PMC2652871 17990498

[40] Baumgartner WA Jr, Lu Z, Johnson HL, Caporaso JG, Paquette J, et al (2008) Concept recognition for extracting protein interaction relations from biomedical text. Genome Biol 9 Suppl 2: S9. 18834500 10.1186/gb-2008-9-s2-s9 PMC2559993

[41] Jin F, Huang M, Lu Z, Zhu X (2009) Towards automatic generation of gene summary. In: Proceedings of the BioNLP 2009 Workshop. Boulder, Colorado: Association for Computational Linguistics. pp. 97–105.

[42] Pestian JP, Brew C, Matykiewicz P, Hovermale D, Johnson N, et al.. (2007) A shared task involving multi-label classification of clinical free text. In: Proceedings of BioNLP 2007. Association for Computational Linguistics.

[43] Hersh W, Voorhees E (2008) TREC genomics special issue overview. Information Retrieval

[44] Kaner C, Nguyen HQ, Falk J (1999) Testing computer software. 2nd edition. John Wiley and Sons

[45] Cohen KB, Baumgartner Jr WA, Hunter L (2008) Software testing and the naturally occurring data assumption in natural language processing. In: Software Engineering, Testing, and Qual-ity Assurance for Natural Language Processing. Columbus, Ohio: Association for Computational Linguistics. pp. 23–30.

[46] Johnson HL, Cohen KB, Hunter L (2007) A fault model for ontology mapping, alignment, and linking systems. In: Pacific Symposium on Biocomputing. World Scientific Publishing Company. pp. 233–244. PMC2516303 17990495

[47] Hearst M, Divoli A, Jerry Y, Wooldridge M (2007) Exploring the effcacy of caption search for bioscience journal search interfaces. In: Biological, translational, and clinical language processing. Prague, Czech Republic: Association for Computational Linguistics. pp. 73–80.

[48] Divoli A, Hearst MA, Wooldridge MA (2008) Evidence for showing gene/protein name suggestions in bioscience literature search interfaces. Pac Symp Biocomput 2008: 568–579. 18229716

[49] Uzuner O, South BR, Shen S, Duvall SL (2011) 2010 i2b2/VA challenge on concepts, assertions, and relations in clinical text. J Am Med Inform Assoc 18: 552–556. 21685143 10.1136/amiajnl-2011-000203 PMC3168320

[50] Cohen KB, Demner-Fushman D (forthcoming) Biomedical natural language processing. John Benjamins Publishing Company

[51] Cohen KB (2010) Biomedical text mining. In: Indurkhya N, Damerau FJ, editors. Handbook of natural language processing. 2nd edition.

[52] Jackson P, Moulinier I (2002) Natural language processing for online applications: text retrieval, extraction, and categorization. John Benjamins Publishing Company

[53] Nobata C, Collier N, Tsujii J (1999) Automatic term identification and classification in biology texts. In: Proceedings of the fifth Natural Language Processing Pacific Rim Symposium (NLPRS). pp. 369–374.

[54] Blaschke C, Andrade MA, Ouzounis C, Valencia A (1999) Automatic extraction of biological information from scientific text: protein-protein interactions. Proc Int Conf Intell Syst Mol Biol 1999: 60–67. 10786287

[55] Craven M, Kumlien J (1999) Constructing biological knowledge bases by extracting information from text sources. Proc Int Conf Intell Syst Mol Biol 1999: 77–86. 10786289

[56] Friedman C, Kra P, Yu H, Krauthammer M, Rzhetsky A (2001) GENIES: a natural-language processing system for the extraction of molecular pathways from journal articles. Bioinformatics 17: S74–S82. 11472995 10.1093/bioinformatics/17.suppl_1.s74

[57] Rzhetsky A, Iossifov I, Koike T, Krauthammer M, Kra P, et al (2004) Geneways: a system for extracting, analyzing, visualizing, and integrating molecular pathway data. J Biomed Inform 37: 43–53. 15016385 10.1016/j.jbi.2003.10.001

