# Supplementary material for: Chapter 16: Text Mining for Translational Bioinformatics
Source: PLoS Comput Biol. 2013 Apr 25;9(4):e1003044. doi: 10.1371/journal.pcbi.1003044 (PMC3635962; doi:10.1371/journal.pcbi.1003044)
Supplement: Text S1 — Answers to Exercises. (DOCX) [file pcbi.1003044.s001.docx]

# Text S1: Answers to Exercises

Suggestions for solving questions 1-2 are included in the Exercises themselves.

1. Obtain a copy of a patient record collection from the i2b2 National Center for Biomedical Computing (see e.g. [49]). Download the MetaMap application or API and run it over a set of ten discharge summaries. Use Google to find the current links for the i2b2 data sets and for downloading MetaMap. Note that using the MetaMap application will require writing code to extract results from the MetaMap output file, while using the API will require writing your own application. Which outputs might you consider to identify phenotypes that could be relevant for your research interests?

2. Obtain a collection of 1,000 PubMed abstracts by querying with the terms gene and mutation and downloading the 1,000 most recent. Run the EMU mutation extractor (<http://bioinf.umbc.edu/EMU/ftp>) or a similar tool on them. What genotypes can you identify in the output?

3. A researcher has a collection of 10,000 documents. She wants to retrieve all documents relevant to pulmonary hypertension. The collection contains 250 documents that are relevant to pulmonary hypertension. An information retrieval program written by a colleague returns 100 documents. 80 of these are actually relevant to pulmonary hypertension. What is the precision, recall, and F-measure for this system?

*The program returned the following numbers of true positives, false positives, and false negatives:*

*True positives: 80 (number actually relevant)*

*False positives: 20 (number returned minus number actually relevant, or 100 - 80)*

*False negatives: 170 (relevant documents in the collection minus number of those returned, or 250 - 80)*

*So, precision is:*

*TP / (TP + FP) = 80 / (80 + 20) = 0.8*

*Recall is:*

*TP / (TP + FN) = 80 / (80 + 170) = 0.32*

*F-measure at β = 1 is (2 * P * R) / (P + R) = (2 * 0.8 * 0.32) / (0.8 + 0.32) = 0.46*

4. Explain the difference between *descriptive linguistic rules* and *prescriptive linguistic rules*. Be sure to say which type text mining is concerned with.

*Prescriptive rules place arbitrary restrictions on language use. They do not reflect actual human linguistic behavior, but rather prescribe or forbid particular linguistic forms. In contrast, descriptive rules attempt to provide descriptions of actual human linguistic behavior. The notion of whether or not that behavior is “correct” has no meaning in descriptive rules; the rules themselves are correct if they accurately reflect human language and are incorrect if they do not accurately reflect human language. Text mining is concerned only with descriptive rules.*
